# *“Respect my opinion and I'll respect yours!”*: Exploring the challenges, concerns, and informational needs of vaccine‐hesitant caregivers and pregnant women in the Philippines

**DOI:** 10.1002/puh2.105

**Published:** 2023-07-14

**Authors:** Mark Donald C. Reñosa, Vivienne Endoma, Johanna Beulah Sornillo, Thea Andrea Bravo, Jhoys Landicho‐Guevarra, Mila Aligato, Jeniffer Landicho, Bianca Joyce Sornillo, Maria Paz Demonteverde, Catherine Silvestre, Carol Malacad, Lourdes Pambid, Jimena Llopis, Cecilia Francisco, Marianette T. Inobaya

**Affiliations:** ^1^ Department of Epidemiology and Biostatistics Research Institute for Tropical Medicine – Department of Health Muntinlupa Philippines; ^2^ Heidelberg Institute of Global Health Ruprecht‐Karls‐Universität Heidelberg Heidelberg Germany; ^3^ Save the Children Philippines Quezon City Philippines; ^4^ Center for Utilizing Behavioral Insights for Children Save the Children International Melbourne Australia

**Keywords:** health policy, health system, Philippines, immunization, policymakers, vaccine hesitancy, vaccines

## Abstract

**Background:**

Despite the instrumental role of vaccines, public confidence is declining, and anti‐vaccine movements have increased worldwide. With the goal of informing policy decisions and the development of interventions, we explore the concerns and challenges related to vaccine uptake (of childhood, maternal, and COVID‐19 vaccines) among parents or caregivers of under‐two children and pregnant women in urban and rural communities in the Philippines.

**Methods:**

Between May and June 2022, we conducted combined in‐person and online interviews with purposively selected caregivers and pregnant women in the Calabarzon region (Naic, Cavite and Tanay, Rizal), and National Capital Region (Pasay and Muntinlupa Cities), Philippines. A total of 43 interviews were completed, audio‐recorded, transcribed, and analyzed according to the tenets of hermeneutic phenomenology.

**Results:**

Our results, grounded on the social ecological model, underlined the interplays of contextual or logistical challenges on vaccine uptake, respondents’ overarching concerns about vaccines, and their informational needs that affected their vaccine decision‐making. Respondents described that the long‐established maternal and childhood vaccines no longer represent a high‐risk concern but highlighted their fears and hesitancy particularly to newer vaccines.

**Conclusion:**

Our findings underscore the multilayered challenges in vaccine decision‐making among caregivers and pregnant women. The volatility of vaccine decision‐making necessitates rethinking the current immunization process, recalibrating the health workforce, and reinvigorating the health information delivery channels for more inclusive and responsive health care.

## BACKGROUND

Vaccine hesitancy (VH), which is the delay in accepting or refusing vaccines despite the availability of vaccination services, has been widely recognized as having a major influence on the success of immunization programs across the globe [1]. The VH continuum is multifaceted; parents who are reluctant to vaccinate their children may choose to postpone vaccinations, deny some vaccines while agreeing to others, or accept vaccines in line with the recommended schedule [1, 2]. Scholars proposed the 5C model of VH (which expands the WHO Strategic Advisory Group of Experts on Immunization's 3C model [1]) outlining the plethora of factors surrounding the phenomenon: complacency (concerning the severity of vaccine‐preventable illness), confidence (in vaccines and the broader health system), constraints (psychological, financial, or structural barriers), calculation (the degree to which individuals search for information about vaccines), and collective responsibility (a willingness to work together to ensure the health of the population) [3]. Scholars recommended using this 5C model while taking into account the determinants separated into three groups: environmental impacts, individual or group influences, and vaccine influences [1, 4]. This global situation varies between and within countries, which may disproportionately affect the already weak immunization structures of low‐ and middle‐income countries (LMICs) [5].

Numerous global studies have investigated VH [6, 7, 8, 9], and several studies in Europe and in North America have concluded that concerns about vaccine safety, lack of trust in government and health care institutions, and an increase in anti‐vaccine sentiment on social media all contributed to decline in vaccination rates [10, 11, 12, 13]. This crucial problem has also been increasing in Asia, Africa, and South America in recent years [14, 15, 16]. Misinformation, mistrust in government and pharmaceutical companies, and religious or cultural views have all led to VH in many nations [17, 18, 19]. Some researchers have also concluded that VH was more prevalent in LMICs, where access to vaccines and health care may be limited and misinformation was more widespread [20, 21]. A systematic review synthesized available evidence on the existing vaccination concerns in LMICs, which included issues with the harmful effects of vaccines, issues with mistrust, and problems with access or health systems [22]. A recent study has also examined the role of fathers or household heads, and how power relations and gendered expectations negatively affect vaccine decision‐making [23].

The Philippines, in particular, is experiencing an erosion of public trust in vaccines [6, 24]. A study in 2018 concluded that confidence in vaccines has plummeted from 93% in 2015 who “strongly agree” on the importance of the vaccines to 32% in 2018 [24]. In addition, a study highlighted the decline in measles vaccination rates from 88% in 2014 to 55% in 2018 [25]. The steep declines were attributed to the controversy surrounding the dengue vaccine (Dengvaxia) and the resulting political turmoil in the country [25, 26]. Aside from significant drops in immunization coverage, one study revealed a significant delay in vaccination among Filipino infants (approximately 39.3% of the infants had delayed completion of vaccination) [27]. The Philippines is currently one of the 10 LMICs with the highest proportion of unvaccinated or under‐vaccinated children globally [28].

Although there is already a VH spread in the country, the COVID‐19 pandemic has added to the health care system's challenges. A recent qualitative study conducted in urban and rural Philippines found that due to community lockdowns and fear of attending health care facilities, caregivers delayed or refused to vaccinate their children [29]. Although various efforts have been made to increase the supply of COVID‐19 vaccines in the country, there have been reports that Filipinos have decided to wait for better options because the majority of the initial doses are produced in China and are not considered as effective as Pfizer BioNtech or Moderna [30]. In addition, Filipino researchers reported that caregivers were fearful of novel vaccines, such as the Dengvaxia and the COVID‐19 vaccines, which were perceived as more harmful than long‐established childhood vaccines [29]. Several scholars have also reiterated how the social trauma caused by the Dengvaxia controversy contributes to Filipinos’ VH toward COVID‐19 vaccines [31, 32, 33]. Studies of VH among parents or caregivers of small children are limited, focused on the early phases of COVID‐19 vaccination, and excluding the perspectives and experiences of other household members [29, 34].

Aside from the recommended maternal immunization, there was also evidence that pregnant women were hesitant to receive COVID‐19 vaccines [35, 36]. A survey of 16 countries (the Philippines was one of the study sites) in 2021 concluded that there were substantial disparities in the adoption of the COVID‐19 vaccine among pregnant women and mothers of small children [37]. The report affirmed that the vaccination rates in the Philippines were generally high, at over 60% among pregnant women and over 75% among mothers of children, relative to other countries surveyed [37]. While confidence in and adoption of vaccines as a whole improved significantly after the Dengvaxia controversy [6], there were still concerns about children not receiving life‐saving vaccines [8]. In light of the potential cascading effects of their eventual opposition to their newborn's immunization, it is essential to investigate VH from the perspectives of pregnant women along with caregivers of small children.

In this paper, we describe the challenges and concerns of caregivers of under‐two children and pregnant women toward childhood, maternal, and COVID‐19 vaccines. The findings are essential for identifying specific health behaviors that could assist policymakers and program managers in developing a culturally tailored intervention to promote vaccine uptake in urban and rural Filipino communities.

## METHODS

### Study design and settings

This study employed a qualitative research design and utilized data collection techniques such as in‐depth interviews (IDIs), records review, and observation in selected cities and municipalities in the Philippines. The Republic of the Philippines is an archipelago in Southeast Asia consisting of more than 7000 islands divided into 17 administrative regions [38]. The most recent Demographic Health Survey indicated that there is a wide variation in immunization rates, and the country has never reached a 95% baseline immunization rate [38].

**FIGURE 1:**
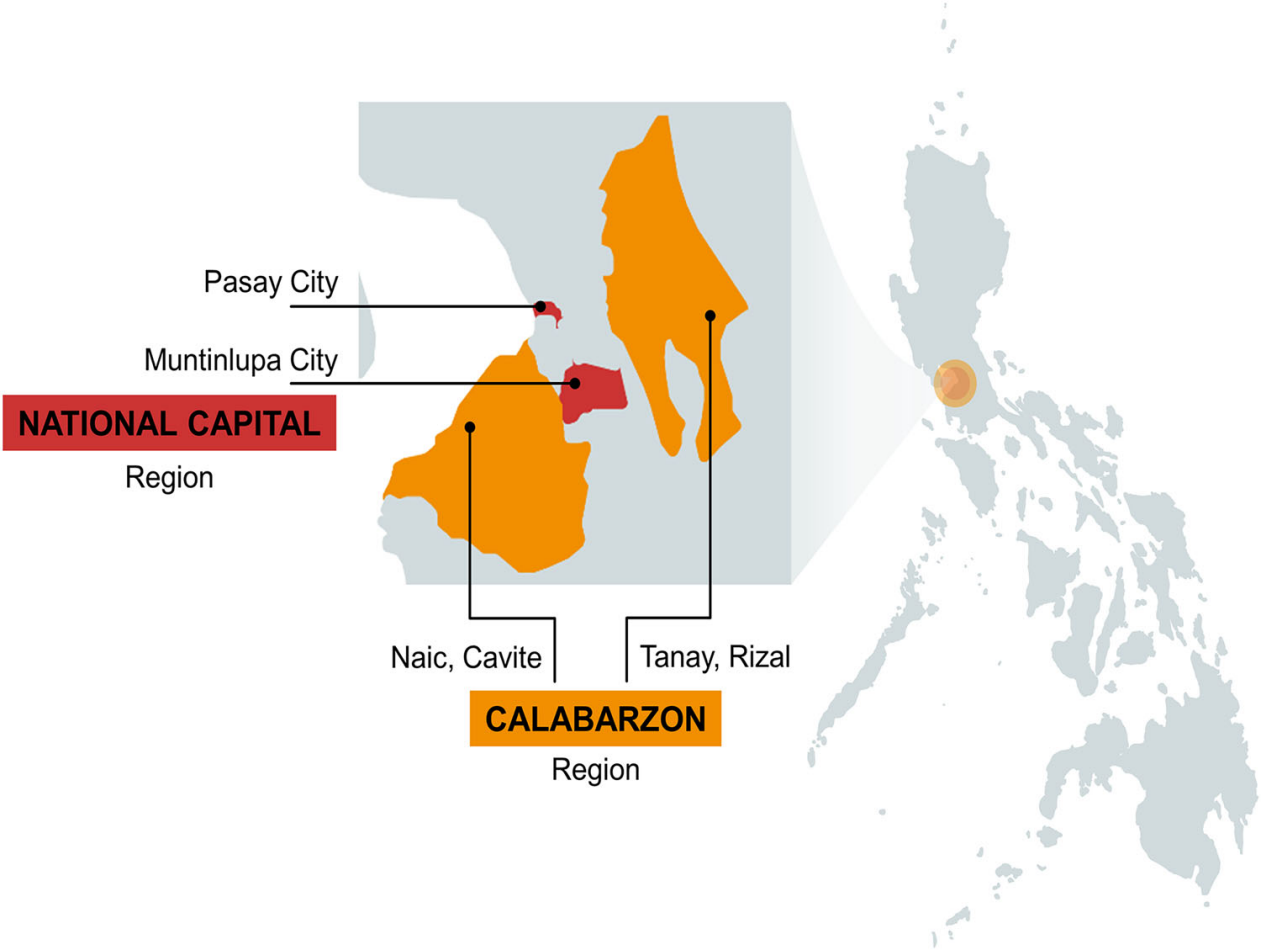
Map of the Philippines, showing the four study areas in the National Capital Region and Calabarzon region. *Source*: Created with mapchart.net.

The study was conducted in two regions in the country that have had recent measles outbreaks and have low immunization coverage, namely, the National Capital Region (NCR) and the Calabarzon (Region IV‐A) region. In particular, we deliberately selected the cities of Pasay and Muntinlupa for NCR, and Naic, Cavite and Tanay, Rizal in the Calabarzon region to accommodate both urban and rural conditions, to capture distinct and diverse sociodemographic factors (i.e., different in terms of wealth indices, household characteristics, and number of children), and to account for possible variations in the health care facility experiences with vaccinations (i.e., among the areas included in the pilot rollout of Dengvaxia vaccines as part of school‐based immunization) (see Figure 1) [39]. Additionally, these regions are to account for the extremely high population mobility in informal settlement areas, which makes vaccine follow‐up challenging [40].

#### Data collection

**FIGURE 2:**
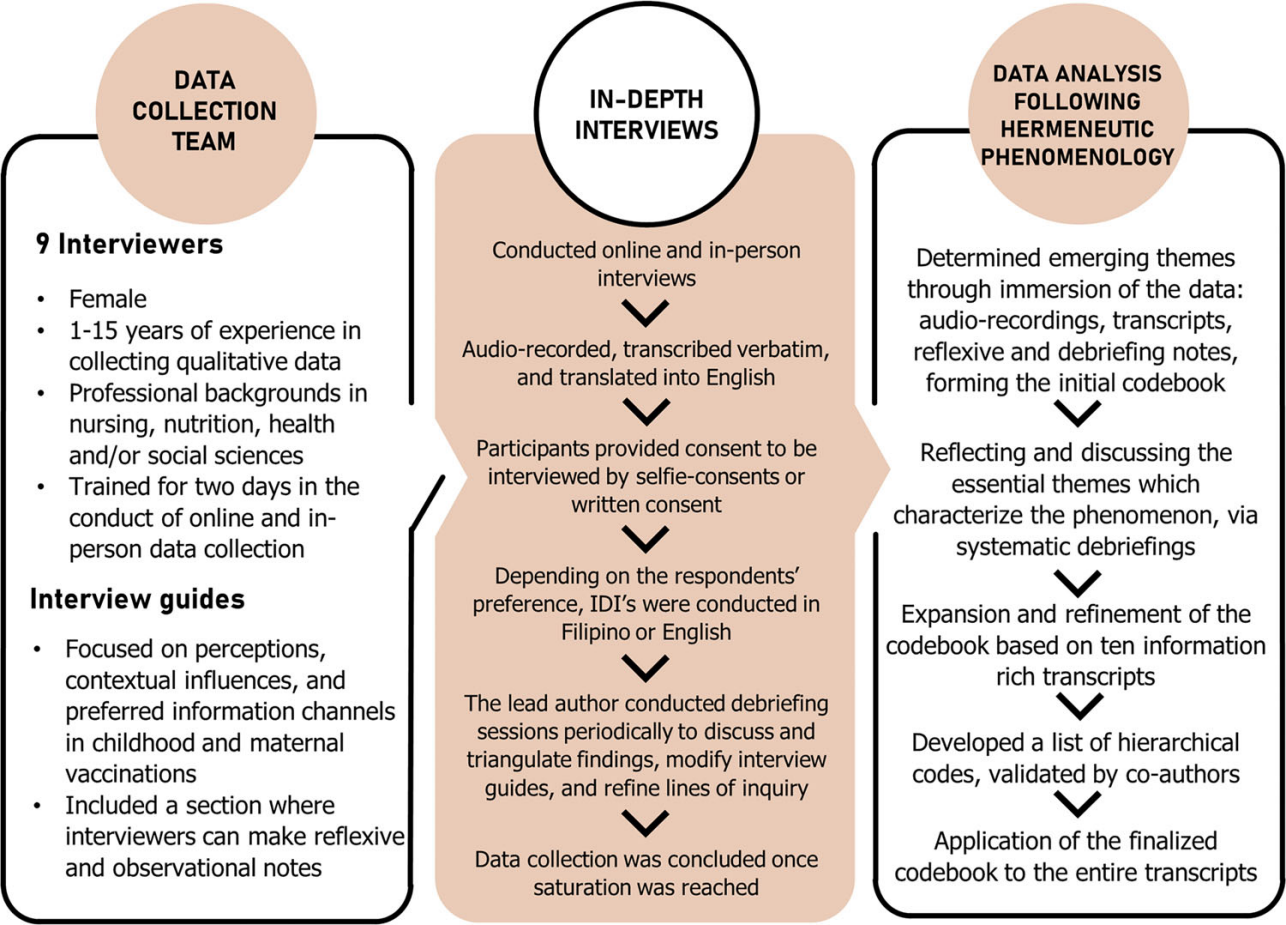
Description of study recruitment, data collection, and analysis.

In April 2022, nine members of the data collection team were trained to collect qualitative data using semi‐structured interview guides. These tools included respondent information sheets and interview guides with a section for them to take reflexive and observational notes. The instruments were pretested and revised as necessary. All interviewers are fluent in Filipino and English, with 1–15 years of qualitative interviewing experience, and had at least a college degree (see Figure 2).

Between May and June 2022, we conducted semi‐structured IDIs with parents or caregivers of under‐two children (including those with disabilities) and pregnant women to understand their perceived challenges and concerns on vaccines, including how the current pandemic posed both challenges and opportunities in vaccine uptake. We used purposive sampling to gain a maximum range of perspectives and depths of information [41]. We included those who are vaccine‐hesitant (individuals who delay or refuse vaccines) and/or individuals who have strong opinions about vaccines in general. We followed Creswell's (2004) recommendation to have 20–40 respondents as this seemed feasible to achieve the study goals [42]. However, these estimates are not indicative as we have followed the guiding principle of data saturation or the point at which no new information is obtained and redundancy is achieved [43]. Written informed consent was obtained from all study respondents before the interviews began. Data collection concluded when saturation was reached.

Amid the pandemic and due to the restrictions surrounding the national elections in the first weeks of May 2022, we shifted our training and data collection procedures at one study site (i.e., Muntinlupa City) to remote delivery and employed in‐person data collection for the next three sites. We followed guidelines for online data collection approach and developed a field manual to ensure data quality and trustworthiness [44]. Depending on the respondent's preference, IDIs were conducted individually in Filipino or English at a private place, and audio or online recordings were made before being simultaneously transcribed and translated into English.

#### Data analysis

All interviews recorded on tape or online were transcribed verbatim by trained data transcribers. Precision and accuracy of narratives and observations were guaranteed by having other members of the research team review and verify the transcriptions. The data transcribers followed the preparation and transcription protocol and principles by McLellan, MacQueen, and Neidig (2003) [45].

The data analysis followed the tenets of hermeneutic phenomenology and was guided by van Manen's data analysis framework [46, 47]. Systematic debriefing sessions were conducted periodically throughout data collection to discuss and triangulate findings, modify interview guides, refine lines of inquiry [48], and determine when we had reached data saturation. The themes that emerged from debriefings or covered in interview guides formed the basis of a codebook. An inductive approach complemented this deductive style: As new themes emerged from the data, they were added to the codebook and applied to the entire dataset. To demonstrate the scope and diversity of themes throughout respondents’ narratives, we framed our findings around common themes. The initial findings highlighted multiple levels of vaccination logistical challenges and layers of individual vaccine decision‐making struggles. Later analysis led to the application of the social ecological model, which reflected the connections and relationships between each theme. Coding was done with NVivo 12 Pro (QSR International Pty Ltd. Version 12, 2018).

#### Reflexivity

Reflexivity is primarily about acknowledging where and how the researcher's previous experiences may have influenced any stage of the research process [49]. The lead author (MDCR) and the members of the data collection team (VE, TAB, JG, MA, JL, BJS, MPD, CS, and CM) are all Filipino nationals currently employed at the Research Institute for Tropical Medicine, the research arm of the Department of Health (DOH), Philippines. We recognize that health care professionals wield considerable sway in communities and that the DOH adopted a prominently paternalistic stance during the COVID‐19 pandemic. We also acknowledge that our roles and prior experience in conducting IDIs may have shaped the way the lead author and the data collection team collected and analyzed the data. The previous involvement of the lead author and some members of the team (MA, JG, JL, TAB, and VE) while working on VH‐related studies have provided foresight in the process of questioning and probing, which led to a comprehensive reflection on to the respondents’ challenges and concerns in vaccines.

As we reflect on our experiences, we understood that the way we perceive the world is different from others’ perceptions and that reality is subjective and multiple. This is why we used hermeneutic phenomenology, an approach that required us to cocreate experiences with respondents’ data in order to get a holistic, deeper understanding of the phenomenon [46, 47]. We believe that this phenomenology approach offered a means to elucidate and elaborate upon our and the respondents’ mutually enriching experiences. In turn, we acknowledge the importance of looking at the complexity of views rather than narrowing the meanings into a few categories of ideas.

We considered the respondents’ feelings throughout the entire data collection procedure. The respondents were briefed beforehand on what to expect from the interview and assured that if they ever felt uncomfortable at any time throughout the process, they were allowed to end it. The social desirability biases may have been eliminated as a result of these constant reassurances. Furthermore, other members of the team, whose previous involvements included studies in child and maternal health, leprosy, rabies, nutrition, and mental health, have contributed to reducing bias due to the minimized exposure to studies concerning VH. We also included systematic debriefing sessions on the interviewer's emotions during the entire data collection process to reduce the contamination of data. These layered processes have aided in making our data reliable, improved our understanding of the influences of contextual forces, and sparked the emergence of new insights.

#### Ethical considerations

This study complied with the principles embodied in the Declaration of Helsinki (2013), the Philippines National Ethical Guidelines for Health Research and Health‐Related Researches (2017), and the applicable regulations of the DOH and respective local governments. In addition, the RITM‐Institutional Review Board reviewed and approved this protocol (approval no.: 2022‐03) prior to its implementation.

## RESULTS

A total of 43 interviews were conducted with parents or caregivers of children under‐two and pregnant women. Three respondents refused to participate. Reasons included the spouse's refusal to participate (*n* = 2), and one gave no specific reason. Tables 1 and 2 show the demographic characteristics of the respondents. A majority of interviewed parents or caregivers of under‐two children were female (95.2%) and had completed at least primary education (85.7%). More than half of the parents or caregivers had at least three or more children (57.1%). Meanwhile, a majority of the pregnant women were in the second trimester of pregnancy (54.6%) and were not first‐time mothers (72.7%).

**TABLE 1:** Demographic profiles of parents and caregivers who had vaccine concerns in the Philippines, May–June 2022 (*n* = 21).

Characteristics	*n*	%
Sex		
Male	1	4.8
Female	20	95.2
Civil status		
Single	1	4.8
Married	7	33.3
Live‐in	13	61.9
Age group		
<18 years	0	0.0
18–30 years	13	61.9
31–40 years	5	23.8
41–50 years	3	14.3
Number of children in the household		
1–2	9	42.9
3–4	7	33.3
5 and above	5	23.8
Number of children under‐2 in the household		
1	15	71.4
2	6	28.6
Are all children under‐2 in the household fully vaccinated?		
Yes	8	38.1
No	13	61.9
Highest educational attainment		
None	2	9.5
Primary	10	47.6
High school	6	28.6
Tertiary	2	9.5
Vocational training	1	4.8
Religious affiliation		
Roman Catholic	18	85.7
Christian	2	9.5
Adventist	1	4.8

**TABLE 2:** Demographic profiles of pregnant women who had vaccine concerns in the Philippines, May–June 2022 (*n* = 22).

Characteristics	*n*	%
Pregnancy term		
First trimester	3	13.6
Second trimester	12	54.6
Third trimester	7	31.8
Civil status		
Single	3	13.6
Married	4	18.2
Live‐in	15	68.2
Age group		
<18 years	2	9.1
18–30 years	12	54.6
31–40 years	7	31.8
41–50 years	1	4.5
Number of children in the household		
None	6	27.3
1–2	6	27.3
3–4	6	27.3
5 and above	4	18.1
Highest educational attainment		
Primary	6	27.3
High school	12	54.5
Tertiary	4	18.2
Religious affiliation		
Roman Catholic	19	86.4
Iglesia ni Cristo	1	4.5
Christian	2	9.1

**FIGURE 3:**
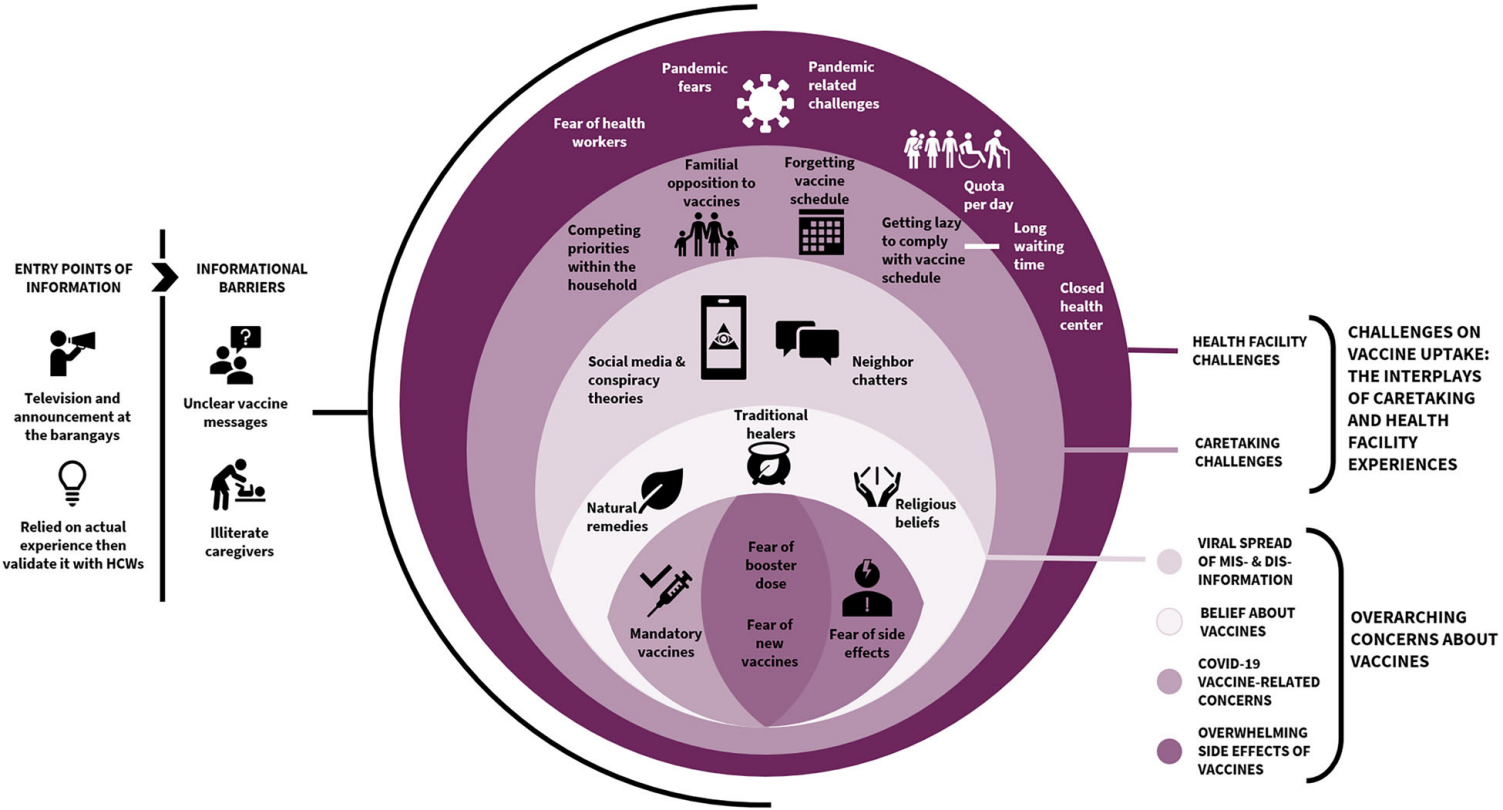
The overarching challenges and concerns of Filipino caregivers and pregnant women on vaccine decision‐making.

To highlight the complex and volatile nature of vaccination narratives evident in our results, we present a model that explores the interactions among contextual challenges, respondents’ internal beliefs and concerns, and information needs within the framework of social ecological model (see Figure 3). The model also illustrates how respondents navigated the challenges of vaccine decision‐making and how the larger vaccine narratives are embedded in overwhelming processes across respondent categories.

Barriers that resonated across respondents included the structural challenges faced in health care facilities, particularly during the COVID‐19 pandemic, and competing household priorities, causing caregivers and pregnant mothers to delay their immunization schedules. Emerging results also indicated that respondents generally accepted vaccinations for children and pregnant mothers. Although there were some legitimate concerns about side effects, there were also feelings of sensitivity to these anticipated vaccine adverse events, either due to personal experiences (and/or the experiences of their friends and family members) or to advice and knowledge provided by health care workers (HCWs) on how to deal with side effects related to vaccination.

All respondents identified COVID‐19 pandemic fears and pandemic‐related challenges as the primary causes of VH. No matter their age or the number of children they had, respondents voiced worries and skepticism regarding the introduction of COVID‐19 vaccines due to the spread of false information in neighborhood chats, social media, and mainstream media. Respondents, especially first‐time mothers, were overwhelmed by the vaccine disinformation as they attempted to sort through the nuanced vaccine information. Respondents from all groups stressed that they still rely on expert advice, such as that given by doctors and nurses in the health centers, despite the widespread dissemination of false information concerning vaccination.

We present the key quotes of each of the themes and subthemes highlighted in Table 3. For each theme, we listed key quotes, followed by the respondent's group (P2 = parents or caregivers of children under‐two; PG = pregnant women), their age, and the number of children as identifiers.

**TABLE 3:** Experiences and perceptions of caregivers and pregnant women on vaccines in the Philippines, May–June 2022.

Themes	Subthemes	Illuminating quotes
**Challenges to vaccine uptake: The interplays of caretaking and health facility experiences**		
Challenges in receiving and following vaccine schedules	*Getting lazy due to competing household priorities*	“Ma'am sometimes because I get lazy especially when there is too much work at home, sometimes I overlooked the time.” (P2, 19 y/o, with 2 children)
	*Forgetting the vaccine schedules*	“Sometimes I missed it [vaccine schedule] … [laugh] because I have many children.” (P2, 23 y/o, with 3 children)
	*Familial opposition to vaccines*	“I get scolded by my mother‐in‐law when I let my child be vaccinated.” (P2, 42 y/o, with 8 children) “When children are vaccinated, they will have fever…that's why my husband is getting mad if our child will be vaccinated…” (P2, 33 y/o, with 5 children)
Health facility challenges: general experiences in getting vaccination services	*Long waiting time, quota per day, and closed health center*	“hmmmm. […] That's how it is in all public centers, you need to go with the queue, or if you want to finish quickly you need to be early, to be entertained or get in the line first so you can get your children be vaccinated quickly.” (PG on trimester, 43 y/o, with 3 children)
	*Fear to health care workers*	“The next day ma'am I was scolded. […] The one who is giving the vaccine to my child [baby crying]… Sometimes, it's embarrassing [!], I felt embarrassed because others can hear it.” (P2, 19 y/o, with 2 children)
	*Pandemic fears*	“There is fear especially when it comes to vaccinations, sometimes at the health center, all the diseases are being seen at the same time … and we don't know their specific disease, oh, of course, it's scary.” (P2, 30 y/o, with 4 children)
	*Pandemic‐related challenges*	“Ah before it's easy, easy because you don't need use facemask, alcohol, and do social distancing. It's so difficult to adjust.” (PG on third trimester, 21 y/o, with 4 children)
**Overarching concerns about vaccines: General acceptance of childhood and maternal vaccination but reluctancy toward COVID‐19 vaccine**		
Overwhelming side effects of vaccines	*Fear of side effects*	“They get a fever, they get sick, so I said, what the heck! the child is in good condition and when the medicine was injected, suddenly my child gets sick […] when I came home, I felt horrible. I blame myself that my baby had a fever again […] I told myself next time, I will never let [my child] go back there.” (P2, 39 y/o, with 6 children)
The viral spread of mis‐ and disinformation	*Neighbor chatters*	“Some people said that COVID is not a real disease, […] it looks like other countries are just destroying us so that they can sell injections. Others said that there is no real disease that COVID seems to be just spreading by other countries.” (P2, 42 y/o, with 8 children)
	*Social Media*	“… I saw it in Facebook that … that the child was just lying down after two days of vaccination and could no longer walk, […].” (P2, 27 y/o, with 3 children)
	*Conspiracy theories*	“Because some people say that that's why they created that disease now because it's supposed to reduce the number of people in the world because there are too many. […] to reduce the number of people … that's what I heard.” (P2, 39 y/o, with 6 children)
Beliefs about vaccines	*Natural remedies*	“I knew my child was healthy so I thought that I can just feed my child or just give my child healthy foods that's what I am thinking […] She [the health care worker] said that my child has the right to be vaccinated, no, she can't convince me […] I just feed them well, you saw that naughty boy, look at his body [pointing to chubby body]” (P2, 19 y/o, with 2 children)
	*Religious beliefs*	“Because the vaccine, during our time, when we studied the bible. […] It says that there will be a time that we will be marked, now if you are among those who do not have a mark, you cannot avail the benefits from the government. […] Which is what's happening right now. I just said, respect my opinion and I'll respect yours!” (P2, 39 y/o, with 6 children)
	*Traditional healers*	“… my husband knows how to do traditional healing. […] he put some smoke and accompanies it with prayers […]” (P2, 22 y/o, with 2 children)
COVID‐19 vaccine‐related concerns	*Fear of new vaccines*	“It's new to my hearing and then was released suddenly, all children had to be vaccinated. I'm scared again that maybe later there will be side effects on my child again …” (P2, 30 y/o, with 4 children)
	*Mandatory vaccination*	“… my husband, the place where he is working for, you need to get vaccinated because it was implemented before no vaccine no work.” (P2, 30 y/o, with 4 children)
	*Fear of the booster dose*	“…we are afraid to get a booster because I have heard that the booster causes death… then you will have short breaths like that, that your body becomes very numb […] so those booster I felt scared to get vaccinated.” (P2, 27 y/o, with 3 children)
**Informational needs**		
Entry points of information	*Relied on actual experience then validate it with HCWs*	“… I usually ask first to, those of my acquaintances who have been vaccinated, those who have already taken the vaccine, ah what are their feelings, if they are ok like that …” (PG on trimester, 30 y/o, with 3 children)
	*Television and announcement at the barangays*	“We really rely on TV. Because isn't it sometimes the baby vaccines are being commercialized as well. Sometimes we find it in the news.” (P2, 30 y/o, with 4 children)
Informational barriers	*Illiterate caregivers*	“…Because I don't have enough education. Sometimes I asked my husband. I tell him to take it, and read it well because he can read, and he understands. Look at this, ask, search for it, and then please read … see if it's good or bad.” (P2, 32 y/o, with 3 children)
	*Unclear vaccine messages*	“… I was confused sometimes in the health center, what is this vaccine? What for? For me, I want to know the name of the vaccine, what it is for… then, for me, I want to also know the side effect.” (P2, 30 y/o with 4 children)

### Challenges to vaccine uptake

#### Challenges in receiving and following vaccine schedules

Vaccines were widely seen by parents and pregnant women as an integral way to safeguard their health, their children, and their community at large. However, there were family‐wide concerns that delayed the required or scheduled vaccination. Respondents said that even though, they were aware of the necessity to visit the health facility, their husbands did not allow them to go after the immunization schedule. Pregnant women, exhausted from caring for their other children, also reported a lack of drive to get up and go for the scheduled vaccination. Some respondents also acknowledged the challenges they faced in deciding whether or not to vaccinate their children as other family members (such as the child's father or in‐laws) were opposed to vaccination, leaving them with no alternative except to refuse vaccination in order to avoid causing strife in the household.

#### Health facility challenges: general experiences in getting vaccination services

Respondents shared extensive anecdotes about their personal experiences getting vaccines, ranging from resigned acceptance of the health care system's realities to disappointments with its infrastructure. Some respondents were denied service at health facilities due to “quotas per day” policies, forcing them to get up early to beat the lines (P2, 27 y/o with one child). In most cases, respondents had to wait at least four hours to see a doctor or get vaccination at the health facilities. Respondents also mentioned instances when they felt embarrassed by the behavior of some HCWs, particularly the nurses and midwives, at the health facilities. Some felt humiliated when criticized in front of other mothers. Some respondents said that they stopped getting vaccinations or went to a different health facility because of the care they received from HCWs.

Respondents felt discriminated against and had trouble following vaccine schedules due to the COVID‐19 pandemic. They have discussed the health facilities’ new mask and social seclusion policies. Some concerns about these new arrangements were unclear and unanswered, making them angry or confused. For instance, a pregnant woman said she felt excluded and discriminated against in the new COVID‐19 waiting rooms that separated the vaccinated from the unvaccinated. In addition, new practices frightened the community: One mother said that she had to hand her infant to an HCW and stand in the health center's doorway. Therefore, she did not see the actual vaccination of her child.

### Overarching concerns about vaccines

#### Overwhelming side effects of vaccines

In general, respondents accepted childhood and maternal vaccination but showed strong resistance to COVID‐19 vaccines. The potential risks associated with vaccines caused widespread fear and anxiety. Fever was one of the most feared side effects in children. The notion that children are too small to withstand the injections has surfaced. Although most respondents were prepared for the potential side effects (either because they had been primed by HCWs or because they had experienced them with their other children or because their mothers mentioned that these side effects were normal), others were still scared to return to the health center for the subsequent doses.

#### The viral spread of misinformation

A majority of the respondents shared how neighbor chatters and social media fueled fears about vaccines. They shared how the spread of fake news on Facebook, YouTube, or TikTok has polarized the general discussion on vaccines. Respondents talked about “*Marites*” (a Filipino slang term for “*Mare*, what is the latest?”) or the neighborhood talks that allow rumors to spread rapidly. Misinformation circulated, not regarding childhood vaccinations, but about COVID‐19 vaccines, which were seen as a means of government control and the pandemic itself as a cover to engage in corrupt activity.

#### Beliefs about vaccines

Some respondents opposed vaccination because of certain religious or cultural beliefs. One respondent mentioned that she would like her children to be exposed to natural remedies rather than having foreign substances injected into her children's bodies. There were also insinuations that some of the vaccinated children were weaker and sicker than those who were unvaccinated. Some also relied more on traditional healers rather than going to the health center. Additionally, some respondents were also guided by their religious beliefs, citing that this COVID‐19 vaccine is part of the greater scheme for human and/or demon control found in the Bible.

#### COVID‐19 vaccine‐related concerns

The introduction of new vaccines, especially COVID‐19 vaccines, was of primary concern to respondents, rather than childhood or maternal vaccines. Respondents reported a widespread apprehension about recent immunizations, which they attributed to the prior Dengvaxia controversy. Parents and pregnant women had strong opinions about the introduction of the COVID‐19 booster doses, claiming that they only consented to the primary doses because they were necessary for them to go to work or go out in public. They saw no need to obtain booster injections as they were previously immunized and they were worried that these additional injections might have “more side effects” (P2, 38 y/o with 3 children).

### Informational needs: entry points and informational barriers

Respondents indicated that they had all the essential knowledge about child and maternal vaccines (i.e., they have actual past experiences and that they have mothers or neighbors to consult with). Concerns solely pertain to data about COVID‐19 vaccines, where there was considerable inconsistency or confusing information. Despite the misinformation, respondents mentioned that they sought knowledge confirmation from HCWs rather than just relying on social media, television, or neighbors’ experiences. Respondents expressed confidence in the government and HCWs, believing that the services they provide are beneficial to the community's health. However, some information provided was difficult to understand, particularly for the illiterate parents.

### Pregnancy and pregnancy‐specific concerns

Although pregnant women generally reflected the challenges and concerns of parents of under‐two children, they emphasized how stressful the various levels of vaccine decisions they faced in their household. This refers to the process of deciding whether to receive maternal vaccines, childhood vaccines, and COVID‐19 vaccines for her and her adolescent children (see Box 1, a case of a pregnant mother).

**Box 1. A story of a pregnant mother facing different layers of household vaccine decision‐making**

Maria (not her actual name), a third‐trimester pregnant mother (with three children and residing in a rural area), revealed her struggles with the decision to vaccinate herself and her family. She began by explaining that she was urged to transfer from the health center to a hospital because the health center was unable to manage the complications of her current pregnancy. Maria mentioned that she has “*manas”* or edema (i.e., during pregnancy, the excess fluid in the body and the strain from the developing uterus can cause swelling in the ankles and feet) and that her condition requires further monitoring at the hospital. She disclosed that she had difficulties getting prenatal check‐ups and that other health centers would not accept her due to her *manas*; as a result, she agreed to midwife‐assisted home birth for a minimal fee (amounting to 2000 Philippine pesos; approximately 40 US dollars).

When Maria was asked about her maternal vaccination status, she mentioned that she had completed her tetanus toxoid shots at the health center and confirmed that she had no problems with the vaccine as it was safe and had no side effects. In contrast to maternal vaccines, she expressed her concerns regarding childhood vaccines, recalling her one child who experienced injection site swelling after receiving a vaccine (upon probing, Maria could not recall the specific vaccine). She panicked and rushed to the health center; however, to her dismay, the health center was closed, forcing her to see the “doctor *sa bayan*” (doctor in the town area). Despite this scary experience, all of her children were fully vaccinated because the doctor assured her that the vaccine side effects are normal and that vaccines are vital for her children's health. However, although she complied with all immunization for herself and her children, she mentioned that the long lines and pandemic‐related challenges in health facilities frequently tested her patience.

When asked about the COVID‐19 vaccine, Maria emphasized that she had not known she was pregnant when she received her first COVID‐19 vaccine, and that she was concerned about the vaccine's potential effects on her unborn child. She shared that her husband was scared of COVID‐19 vaccines, telling her “Who will provide for the family if something happens to me?” This situation also applies to their adolescent child, citing that her child wanted to see the effect of the vaccine on his classmates before he gets an injection. However, this perception changed when the government mandated vaccinations for work and school. “It's not good to force people to get vaccinated, especially when there are side effects,” Maria stated in opposition to the government’s strategy. As a result, she was adamant about receiving COVID‐19 booster doses as it is no longer mandatory. Moreover, Maria's hesitation is profoundly anchored in her chronic mistrust of pharmaceutical companies; she stressed that “they [pharmaceutical companies] are just practicing in developing vaccines.”

## DISCUSSION

Our study outlines the challenges in vaccine decision‐making as described by caregivers and pregnant women in urban and rural Filipino communities. Concerns regarding health facilities centered primarily on resource‐constrained public health structures and the disposition of HCWs, which highlighted the risks of noncompliance or delayed vaccinations. Respondents felt overburdened when making vaccine decisions due to household competing priorities and the viral spread of misinformation. Trusted messengers described are HCWs, particularly the doctors and their household members’ experiences in dealing with vaccine side effects. Notable narratives corroborate a widespread concern regarding the COVID‐19 vaccines, particularly booster doses deemed superfluous and potentially harmful to their overall well‐being.

Several VH studies at national and global levels have emphasized similar health facility challenges and parental concerns [29, 32, 34, 50, 51]. A recent study in the Philippines echoed the same sentiments regarding the rejection of parental concerns by HCWs, leaving the parent scared and embarrassed to voice vaccine‐related concerns [29]. Several studies suggested that HCWs were overburdened, hence the burnout and demotivation phenomenon, reflecting the current tenor in the literature [52, 53, 54], but evidence of retention strategies, incentives, and other professional support is so far limited. Our findings also highlighted how social media altered communication and stoked widespread anxieties that permeated other contexts [55, 56, 57]. Fear‐based narratives are a common source of misinformation on social media, which has increased apathy and low public engagement [18, 58]. Through exaggerated and unfounded rumors and allegations, this infodemic caused fear and terror [59]. In a large cross‐national study examining the impact of social media on VH, results concluded that international disinformation operations were linked to a decline in vaccine coverage globally [60]. Public health has suffered in recent years as a result of the polarization of vaccine dangers and erroneous anti‐vaccine narratives [32, 33, 61, 68]. Social media has changed how individuals communicate globally, despite government efforts to guide the public to trustworthy sources for verified information and updates. Our findings suggest a new method of dissemination, via “TikTok,” a social networking application known for 2‐ to 3‐min videos as an entertainment tool, such as singing, dancing, and cooking, among others. This platform now serves as a petri dish to sprout anti‐vaccination sentiments freely in the general public's echo chambers, despite some studies confirming that vaccine misinformation primarily occurs on Facebook, Twitter, and YouTube [61, 62]. Technology companies have recognized this infodemic and have proposed several maneuvers to track down and remove false and misleading content by working with fact‐checking partners and local governments. For example, the Philippines DOH recently launched a campaign called #ChecktheFAQs, in which social media companies pledged to support the initiative in the fight against COVID‐19 and vaccine misinformation online [63].

Several studies have highlighted how the COVID‐19 pandemic and COVID‐19 vaccinations are fueling the global discussion on VH [64, 65, 66]. Our findings, however, revealed a shift in attitudes: Vaccinations for pregnant women and children no longer appear to pose a threat to parents and pregnant mothers. Although this attitudinal shift warrants further investigation, this phenomenon is indicative of volatility in vaccination confidence [67]. With changing trends in COVID‐19 viral flares, and more information—and misinformation—about vaccine decision‐making, public confidence in vaccines also suffers an uptrend or downtrend [67]. For instance, some studies in the Philippines highlighted how the Dengvaxia controversy has impacted the current acceptance of COVID‐19 vaccines and that these new vaccines could have long‐term consequences [29, 30, 68, 69]. This volatility in vaccine information is pushing the public to turn against new vaccines and turn more to long‐established vaccines.

Similar to the findings across LMICs, challenges in public and community engagement also appear to be strongly driven by the introduction of new vaccines, such as COVID‐19 and dengue vaccines [5]. Our findings showed that risk communication and community engagement are crucial when introducing new vaccines. In the case of COVID‐19 vaccines, our findings suggested that COVID‐19 containment strategies (i.e., implementing “no vaccine, no transportation” or “no vaccine, no entry to public spaces” among others) forced a sudden attempt at mass behavioral change to approve the COVID‐19 vaccination. The impact of the economic recession and the tensions of the lockdown measures have been compounded by limited resources, inaccurate information, and delays in vaccine rollout, which may have contributed to the emergence of doubt, mistrust, and conspiracy theories. During the initial launch, a recent study described the “vaccine brand hesitancy” phenomenon (defined as delays in vaccine uptake due to preferred vaccine brand) as being prominent in the Philippines [34]. Although that sentiment holds truth, Filipinos are forced to stick to vaccines as a passport to get out of domestic confinement.

Our finding concludes the need for more coherent and streamlined policies, through good governance to ensure that the provision and delivery of services are equitable. The responsiveness of health systems to such extreme conditions with greater matching of public expectations and needs impacts overall success. Our study adds to the academic discourse that emphasizes the need for health education programs to not only be educational, interesting, and responsive to the needs of the general public but also to be safeguarded against being utilized by anti‐vaccination movements as a means of deceiving and misinforming the public. Social media analysis should examine how these new trending media applications are contributing to the proliferation of anti‐vaccine sentiments and consequently the decline in vaccine uptake. Further, our findings revealed that COVID‐19 booster doses were thought to be unnecessary, which could possibly be a ticking time bomb for the new variant outbreaks. It is then integral to devise multiple strategies to reach and cover the population and stimulate public interest in simple, empathic, and prosocial messages, and integrated nudging techniques to increase the uptake of COVID‐19 boosters [70].

Our study has limitations: Because vaccination is a controversial topic in the Philippines, the social desirability bias could limit our results. Our attempt to reduce this bias was through establishing rapport and using probing techniques that allowed respondents to openly share their experiences and perceptions. Amid the pandemic and election‐related restrictions, we switched one study site to online data collection. By moving to online platforms, we may have excluded caregivers who lack the mobile device required to participate in the study. Lastly, a majority of our respondents are mothers. This limited our insights into the caregiving practices and perceptions of other important family members, such as fathers and grandparents.

## CONCLUSION

Our findings provide insights into the lived experiences of caregivers and pregnant women on their hesitancies to receive vaccines in rural and urban Philippines. The volatility of vaccine confidence highlights the best and the worst features of our existing health care system. Our findings recommend program managers develop health information materials on vaccines that are adaptive and responsive to the needs and interests of the general public, and designate authorities to enhance transparent, consistent, and coherent public communication to address misinformation. Although some notable challenges are unveiled, it reminds us of what truly matters—that the chances and actions we should take must focus on inclusivity, equity, and responsiveness. These insights could strengthen our shared hope and vision to keep our promise to leave no one behind.

## AUTHOR CONTRIBUTIONS

Mark Donald C. Reñosa performed data analysis and wrote the initial draft of the manuscript. Vivienne Endoma, Thea Andrea Bravo, Jhoys Landicho‐Guevarra, Mila Aligato, Jeniffer Landicho, Bianca Joyce Sornillo, Maria Paz Demonteverde, Catherine Silvestre, and Carol Malacad conducted data collection. Johanna Beulah Sornillo, Jimena Llopis, Lourdes Pambid, Cecilia Francisco, and Marianette T. Inobaya contributed to the report writing and critically reviewed the manuscript. All authors have read and approved the manuscript.

## CONFLICT OF INTEREST STATEMENT

The authors declare no conflicts of interest.

## FUNDING INFORMATION

Australian NGO Cooperation Program of DFAT Australia, including various donors from Save the Children Philippines

## Data Availability

The datasets generated in this study are not publicly available due to the sensitive and personal nature of the information. Data may be available upon request to authors, with restrictions following ethical approval.
